# Effect of dialytic phosphate reduction rate on mortality in maintenance hemodialysis patients: a matched case–control study

**DOI:** 10.1186/s12882-023-03199-x

**Published:** 2023-06-12

**Authors:** Shuixiu Yang, Zongli Diao, Wenhu Liu, Wang Guo

**Affiliations:** 1 Department of Nephrology, Beijing Friendship Hospital, Capital Medical University, Beijing, 100050 China; 2 Blood Purification Center, Guiyang Public Health Clinical Center, Guiyang, 550001 Guizhou China

**Keywords:** Phosphate reduction rate, Maintenance hemodialysis, All-cause death, Cardiocerebrovascular death

## Abstract

**Background:**

Phosphates, similar to urea, are small molecular substances that can be cleared during dialysis. Dialytic phosphate reduction rate (PRR) may, to some extent, be related to the relative amount of phosphates cleared during dialysis. However, few studies have evaluated the associations between PRR and mortality in maintenance hemodialysis (MHD) patients. In this study, we investigated the association between PRR and clinical outcomes in MHD patients.

**Methods:**

This was a retrospective, matched case–control study. Data were collected from the Beijing Hemodialysis Quality Control and Improvement Center. Patients were divided into four groups according to PRR quartile. Age, sex, and diabetes were matched between the groups. The primary outcome was all-cause death, and the secondary outcome was cardiocerebrovascular death.

**Results:**

The study cohort comprised 4063 patients who were divided into four groups according to the PRR quartile: group PRR_1_ (< 48.35%), group PRR_2_ (48.35% — 54.14%), group PRR_3_ (54.14% — 59.14%), and group PRR_4_ (≥ 59.14%). We enrolled 2172 patients (543 in each study group) by case–control matching. The all-cause death rates were as follows: group PRR_1_: 22.5% (122/543), group PRR_2_: 20.1% (109/543), group PRR_3_: 19.3% (105/543), and group PRR_4_: 19.3% (105/543). No significant differences in all-cause and cardiocerebrovascular death rates according to the Kaplan–Meier survival curves were found between the groups (log-rank test, *P* > 0.05). Multivariable Cox regression analysis revealed no significant differences in all-cause and cardiocerebrovascular death rates between the four groups (*P* = 0.461; adjusted hazard ratio, 0.99; 95% confidence interval, 0.97 – 1.02 versus *P* = 0.068; adjusted hazard ratio, 0.99; 95% confidence interval, 0.97 – 1.00, respectively).

**Conclusions:**

Dialytic PRR was not significantly associated with all-cause death and cardiocerebrovascular death in MHD patients.

## Background

The number of people undergoing renal replacement therapy is increasing globally, which has exceeded 2.5 million and is projected to more than double to 5.4 million by 2030 [1]. Mortality in maintenance hemodialysis (MHD) patients is significantly higher than that in the general population, mainly owing to the high risks associated with cardiocerebrovascular disease [2, 3]. And mortality due to cardiovascular disease in dialysis patients is approximately 20 times higher than in a general population [4]. A poor prognosis and numerous risk factors are associated with MHD, and abnormal phosphate metabolism remains an important risk factor for death [5, 6]. Studies in several countries have found that hyperphosphatemia increases the mortality and cardiocerebrovascular events rates in MHD patients [7]. Severely low serum phosphate concentrations and rapid decreases can cause rhabdomyolysis, respiratory failure, lethargy, and consciousness disturbance. Hypophosphatemia during renal replacement therapy may lead to myocardial dysfunction and prolonged respiratory failure [8, 9]. Changes in serum phosphate concentrations ≥ 0.7 mg/dL (1 mg/dl = 0.323 mmol/L) in non-dialysis hospitalized patients were positively associated with in-hospital mortality [10]. Additionally, high phosphate variability resulted in higher mortality and cardiocerebrovascular event rates in MHD patients [11].

Serum phosphate concentrations are difficult to control in MHD patients, and hemodialysis (HD) is an important therapy to remove serum phosphate. Because intracellular phosphate is responsible for the phosphorylation of various proteins, changes between extracellular and intracellular phosphate concentrations can induce mitochondrial toxicity [12] and abnormal cellular signaling, and thus lead to cell and tissue damage [13]. Moreover, elevated extracellular phosphate can enhance mitochondrial membrane potentials by increasing the permeability of the transition pores, leading to superoxide formation and inducing apoptosis [14]. The calculation method for PRR is the same as urea reduction rate (URR) and can be calculated using the following formula: PRR = 100% × (pre-dialysis Phosphate – post-dialysis Phosphate)/pre-dialysis Phosphate. Although some authors believe that PRR can be used to evaluate the clearance of phosphorus during dialysis [15], it is unknown whether PRR, like URR, is associated with the clinical prognosis of MHD patients. In this study, phosphate reduction ratio in dialysis was investigated to explore its relationship with prognosis, evaluated as all-cause and cardiocerebrovascular death, in MHD patients.

## Method

### Study design

This was a multicenter, retrospective, matched, matched case–control study. We extracted and analyzed data from 4063 MHD patients from the Beijing Hemodialysis Quality Control and Improvement Center, which is a large dialysis center in China. This study involving human participants was reviewed and approved by the Bioethics Committee of Beijing Friendship Hospital, Capital Medical University (IRB number: 2022-P2-301– 01). The need for informed consent was waived by the Bioethics Committee of Beijing Friendship Hospital, Capital Medical University due to the minimal risk nature of the study.

Blood samples were collected for biochemical analysis before and immediately after the dialysis session, which were measured using an autoanalyzer.

### Study cohort

Between January 2014 and December 2018, all HD patients registered in the Beijing Hemodialysis Quality Control and Improvement Center were screened for eligibility according to the following inclusion and exclusion criteria.

Inclusion criteria: (1) first dialysis time from January 2014 to December 2018; (2) age: 18 – 80 years; (3) consistent HD for ≥ 6 months; and (4) serum phosphate concentration measured at least every 6 months before and after dialysis, or at least once every 12 months with a follow-up of more than 36 months, with the median phosphate concentration used if phosphate was measured more frequently.

Exclusion criteria: (1) median serum albumin < 30 g/L; (2) median hemoglobin < 90 g/L; and (3) patients with incomplete clinical data.

The patients were divided into four groups according to the PRR quartile during the study period: group PRR_1_ (< 48.35%), group PRR_2_ (48.35% — 54.14%), group PRR_3_ (54.14% — 59.14%), and group PRR_4_ (≥ 59.14%). Data from eligible patients were collected until death, kidney transplant, transfer to peritoneal dialysis, or December 2021.

### Outcomes

After enrollment, the patients were followed retrospectively until an endpoint occurred or until 31 December 2021. The primary outcome of this study was all-cause death, and the secondary outcome was cardiocerebrovascular death. Cardiocerebrovascular death comprised cardiac arrest and death from heart failure, ischemic heart disease, or cerebrovascular disease.

### Case–control matching

We anticipated that the baseline characteristics of the patients in the PRR_1_, PRR_2_, PRR_3_, and PRR_4_ groups would differ significantly. To avoid confounding as a result of these differences, the following variables were matched between the four study groups: sex, age (± 2 years), and diabetes.

### Statistical analysis

The four groups were matched for sex, age (± 2 years), and diabetes, and the outcomes between the groups were compared.

Continuous variables are presented as mean ± standard deviation or median and interquartile range. We analyzed differences in the patients’ characteristics using analysis of variance, the Kruskal–Wallis test, or the Friedman test, as appropriate. Categorical variables were expressed as frequencies and percentages and were compared using the χ^2^ test or Fisher’s exact test.

Two models were used: (1) survival curves were estimated by Kaplan–Meier analysis, and the curves were compared by the log-rank test as a univariable model with four groups divided by the quartile of the serum phosphate reduction rate between pre- and post-dialysis; and (2) multivariable Cox regression with adjustment for categorical variables (sex, diabetes) and continuous variables (age, PRR, pre-dialysis phosphate, URR, hemoglobin, albumin, and intact parathyroid hormone (iPTH) concentrations). *P* values < 0.05 were considered to denote statistical significance, and all statistical analyses were performed using IBM SPSS 25.0 (IBM Corp., Armonk, NY, USA).

## Results

### Study cohort

**Fig. 1:**
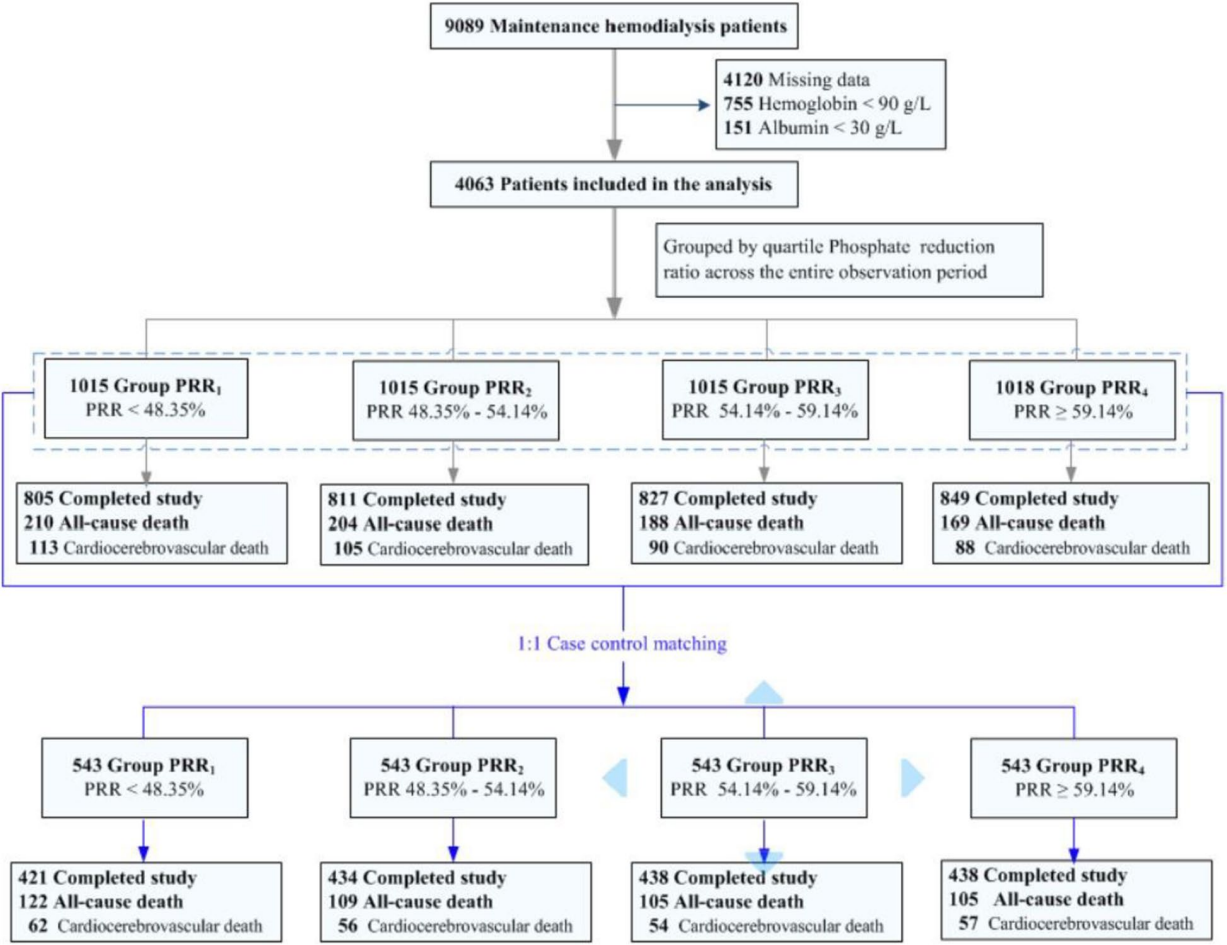
Flow chart of the study. *PRR* phosphate reduction rate

Figure 1 is a flow chart of the study. Between January 2014 and December 2018, 9089 MHD patients were registered with the Beijing Hemodialysis Quality Control and Improvement Center. Of these, 4063 patients were enrolled in this study, namely 1015 in group PRR_1_ (< 48.35%), 1015 in group PRR_2_ (48.35% — 54.14%), 1015 in group PRR_3_ (54.14% — 59.14%), and 1018 in group PRR_4_ ( ≥ 59.14%). Case–control matching resulted in 543 patients in each group.

### Baseline characteristics

The patients’ baseline characteristics in the unadjusted cohort are listed in Table 1. Significant differences (*P* < 0.05) were found between the four groups in age, sex, primary nephropathy, diabetes, and blood laboratory indices (pre-dialysis phosphate, post-dialysis phosphate, PRR, calcium, with no significant differences for hemoglobin, iPTH, albumin, and URR).

**Table 1:** Baseline characteristics of the patients in the unadjusted cohorts

Characteristic	Overall (*n* = 4063)	Group PRR_1_ < 48.35% (*n* = 1015)	Group PRR_2_ 48.35% — 54.14% (*n* = 1015)	Group PRR_3_ 54.14% — 59.14% (*n* = 1015)	Group PRR_4_ ≥ 59.14% (*n* = 1018)	*P* value
Median age (IQR), y	58.0(49.0 – 66.0)	58.0(48.0 – 65.0)	58.1(49.0 – 64.1)	58.2(49.1– 66.0)	60.0(50.0 – 67.0)	< 0.05
Male sex-no. (%)	2529(62.2)	810 (79.8)	732(72.1)	625(61.6)	362(35.6)	< 0.001
Cause of ESRD-no. (%)						< 0.001
Diabetic nephropathy	542(13.3)	155(15.3)	153(15.1)	129(12.7)	105(10.3)	
Glomerulonephritis	876(21.6)	179(16.2)	213(21.0)	233(23.0)	251(24.7)	
Hypertensive nephropathy	519(12.8)	133(13.1)	130(12.8)	123(12.1)	133(13.1)	
Tubulointerstitial nephritis	166(4.1)	43(4.2)	29(2.9)	34(3.3)	60(5.9)	
Others	206(5.1)	38(3.7)	47(4.6)	54(5.3)	67(6.6)	
Unknown	1754(43.2)	467(46.0)	443(43.6)	442(43.5)	402(39.5)	
Diabetes	1788(44.0)	498(49.1)	484(47.7)	441(43.4)	365(35.9)	< 0.001
Pre-dialysis phosphate(mmol/L)^a^	1.80 ± 0.41	1.63 ± 0.40	1.77 ± 0.38	1.86 ± 0.39	1.92 ± 0.39	< 0.001
Post-dialysis phosphate (mmol/L)^a^	0.83 ± 0.21	0.94 ± 0.23	0.86 ± 0.19	0.81 ± 0.17	0.71 ± 0.15	< 0.001
phosphate reduction rate(PRR %)	54.14 (48.35 – 59.14)	44.00 (40.06 – 46.41)	51.43 (50.00 – 52.85)	56.57 (55.37 – 57.80)	62.63 (60.67 – 65.00)	< 0.001
Calcium (mmol/L)^a^	2.21 ± 0.14	2.19 ± 0.14	2.20 ± 0.13	2.21 ± 0.14	2.23 ± 0.13	< 0.001
iPTH (pg/mL)	213.55 (139.80 – 307.73	206.50 (135.70 – 294.60**)**	216.65 (140.46 – 312.85)	215.80 (142.05 – 311.93	214.45 (142.08 – 308.32)	0.156
Hemoglobin (g/L)^a^	112.88 ± 7.20	113.01 ± 8.06	112.95 ± 7.47	112.86 ± 6.85	112.70 ± 7.20	0.759
Albumin (g/L)^a^	39.51 ± 2.96	39.66 ± 3.19	39.44 ± 3.20	39.52 ± 2.75	39.43 ± 2.68	0.291
Urea reduction rate (URR %)^a^	66.86 ± 5.82	66.60 ± 5.93	66.83 ± 6.21	67.06 ± 5.85	66.97 ± 5.82	0.330

Normally-distributed data are presented as means ± SDs, and non-normally distributed data as medians

*PRR* phosphate reduction rate between pre- and post-dialysis, *URR* urea reduction rate, *ESRD* end-stage renal disease, *iPTH* intact parathyroid hormone, *IQR* interquartile range, *SD* standard deviation

^a^Presented as means ± SDs

Case–control matching was performed for all four groups. The matched factors were sex, age (± 2 years), and diabetes history. After matching, the patients’ baseline characteristics, including age, sex, and diabetes, were comparable between the groups; 2172 cases were matched successfully in the four groups, and each group comprised 543 patients. The baseline characteristics of the patients in the matched cohorts are listed in Table 2. We found significant differences (*P* < 0.001) between the four groups for pre-dialysis phosphate, post-dialysis phosphate, and PRR.

**Table 2:** Patients’ baseline characteristics in the matched cohorts

Characteristic	Overall (*n* = 2172)	Group PRR_1_ < 48.35% (*n* = 543)	Group PRR_2_ 48.35% — 54.14% (*n* = 543)	Group PRR_3_ 54.14% — 59.14% (*n* = 543)	Group PRR_4_ ≥ 59.14% (*n* = 543)	*P* value
Median age (IQR), y	59.0(51.0 – 66.0)	59.0(50.0 – 66.0)	59.0(51.0 – 66.0)	59.0(51.0 – 66.0)	59.0(51.0 – 66.0)	0.934
Male sex-no. (%)	1380(63.5)	345(63.5)	345(63.5)	345(63.5)	345(63.5)	1.000
Cause of ESRD-no. (%)						0.479
Diabetic nephropathy	315(14.5)	82(15.1)	76(14.0)	80(14.7)	77(14.2)	
Glomerulonephritis	430(19.8)	102(18.8)	106(19.5)	107(19.7)	115(21.2)	
Hypertensive nephropathy	282(13.0)	70(12.9)	74(13.6)	74(13.6)	64(11.8)	
Tubulointerstitial nephritis	76(3.5)	28(5.2)	16(2.9)	11(2.0)	21(3.9)	
Others	92(4.2)	16(2.9)	25(4.6)	22(4.1)	29(5.3)	
Unknown	977(45.0)	245(45.1)	246(45.3)	249(45.9)	237(43.6)	
Diabetes	1020(47.0)	255(47.0)	255(47.0)	255(47.0)	255(47.0)	1.000
Pre-dialysis phosphate (mmol/L)^a^	1.79 ± 0.41	1.61 ± 0.39	1.74 ± 0.38	1.86 ± 0.39	1.94 ± 0.41	< 0.001
Post-dialysis phosphate (mmol/L)^a^	0.82 ± 0.20	0.92 ± 0.23	0.84 ± 0.18	0.81 ± 0.17	0.72 ± 0.16	< 0.001
phosphate reduction rate(%)	54.13 (48.32 – 59.14)	44.09 (40.23 – 46.43)	51.46 (49.88 – 52.90)	56.47 (55.24 – 57.75)	62.16 (60.40 – 64.45)	< 0.001
Calcium (mmol/L)^a^	2.21 ± 0.14	2.21 ± 0.14	2.20 ± 0.13	2.21 ± 0.14	2.22 ± 0.13	0.550
iPTH (pg/mL)	209.18 (137.87–306.59)	201.00 (132.56–296.60)	213.50 (140.65–306.43)	216.50 (139.0–323.15)	207.90 (135.30–303.25)	0.255
Hemoglobin (g/L)^a^	112.68 ± 7.11	112.27 ± 7.93	112.71 ± 6.97	112.81 ± 6.98	112.93 ± 6.49	0.450
Albumin (g/L)^a^	39.41 ± 3.10	39.47 ± 3.27	39.25 ± 3.44	39.40 ± 2.85	39.50 ± 2.80	0.562
Urea reduction rate (%)^a^	66.77 ± 5.91	66.60 ± 5.99	66.79 ± 6.29	66.85 ± 5.66	66.82 ± 5.67	0.889

Normally distributed data are presented as means ± SDs and non-normally distributed data as medians

*PRR* phosphate reduction rate between pre- and post-dialysis, *URR* urea reduction rate, *ESRD* end-stage renal disease, *iPTH* intact parathyroid hormone, *IQR* interquartile range, *SD* standard deviation

^a^Presented as means ± SDs

### Primary outcome

**Fig. 2:**
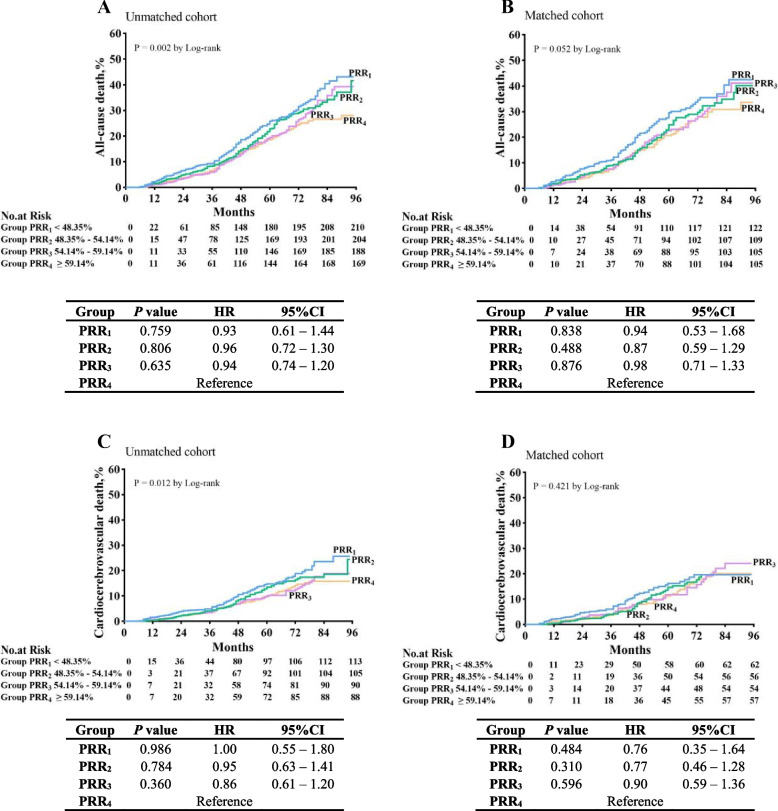
Cumulative event rates. Cumulative event rates for all-cause death associated with PRR among MHD patients in the unmatched (**A**) and case–control matched cohorts (**B**). Cumulative event rates for cardiocerebrovascular death associated with PRR among MHD patients in the unmatched (**C**) and case–control matched cohorts (**D**). *P* values are derived from the univariable analysis. Hazard ratios are based on multivariable Cox regression analyses. *PRR* phosphate reduction rate between the pre- and post-dialysis, *CI* confidence interval, *MHD* maintenance hemodialysis

In the unmatched cohort, the all-cause death rates were as follows: group PRR_1_: 20.7% (210/1015), group PRR_2_: 20.1% (204/1015), group PRR_3_: 18.5% (188/1015), and group PRR_4_: 16.6% (169/1018). Kaplan–Meier survival curves according to study group are shown in Fig. 2A. Significant differences (log-rank test; *P* = 0.002) in the all-cause death rates were found between the four groups. Multivariable Cox regression analysis revealed no significant difference in the all-cause death rates between the four groups (*P* = 0.060; hazard radio (HR), 0.98; 95% confidence interval (CI), 0.96 – 1.00).

In the matched cohort, the all-cause death rates were as follows: group PRR_1_: 22.5% (122/543), group PRR_2_: 20.1% (109/543), group PRR_3_: 19.3% (105/543), and group PRR_4_: 19.3% (105/543). The cumulative all-cause death rates are shown in Fig. 2B. As shown in the Kaplan–Meier survival curves (log-rank test; *P* = 0.052) and the results of the multivariable Cox regression analysis (*P* = 0.461; adjusted HR, 0.99; 95% CI, 0.97 – 1.02), there was no significant difference in the all-cause death rates between the four groups. Multivariate Cox analysis showed that in addition to PRR, hemoglobin, serum calcium, and albumin were also significantly associated with all-cause mortality rate of matched patients. There was no significant correlation between pre-dialysis phosphorus, URR, iPTH and all-cause mortality rate.

### Secondary outcome

In the unmatched cohort, the cardiocerebrovascular death rates were as follows: group PRR_1_: 11.1% (113/1015), group RRP_2_: 10.3% (105/1015), group PRR_3_: 8.9% (90/1015), and group PRR_4_: 8.6% (88/1018). Kaplan–Meier survival curves according to study group are shown in Fig. 2C. Significant differences (log-rank test; *P* = 0.012) in the cardiocerebrovascular death rates were found between the four groups; the death rate in group PRR_1_ was highest. Multivariable Cox regression analysis showed significant differences between the four groups (*P* = 0.001; HR, 0.98; 95% CI, 0.97 – 0.99).

In the matched cohort, the cardiocerebrovascular death rates were as follows: group PRR_1_: 11.4% (62/543), group PRR_2_: 10.3% (56/543), group PRR_3_: 9.9% (54/543), and group PRR_4_: 10.5% (57/543). Kaplan–Meier survival curves for each group are shown in Fig. 2D. No significant differences were found between the four groups in Kaplan–Meier survival analysis (log-rank test; *P* = 0.421), although the PRR1 group still had the highest mortality. The results of the multivariable Cox regression analysis were consistent with this finding (*P* = 0.068; adjusted HR, 0.99; 95% CI, 0.97 – 1.00). The result of multivariate Cox analysis revealed that in addition to PRR, hemoglobin, serum calcium, and albumin were also significantly associated with cardiocerebrovascular death rate of matched patients. There was no significant correlation between pre-dialysis phosphorus, URR, iPTH and cardiocerebrovascular death rate.

## Discussion

In this study, we matched for age, sex, and diabetes at baseline between four PRR groups to eliminate the cofounding bias effects of these known variables. The results showed that PRR was not significantly correlated with all-cause death and cardiocerebrovascular death in MHD patients.

Hyperphosphatemia may lead to a variety of adverse complications in MHD patients, and most previous studies have shown that hyperphosphatemia increases the risk of death. The current Kidney Disease: Improving Global Outcomes (KDIGO) guidelines recommend that the control of serum phosphate be based mainly on the "3D principle", that is, limiting dietary phosphate intake, taking phosphate binders, and ensuring adequate dialysis [16]. Conventional 4-h HD removes only approximately 800 – 1000 mg of phosphate during each dialysis session. Eighty-five percent of the phosphate in the body exists in bone and teeth in the form of hydroxyapatite, and only 1% exists in extracellular fluid, such as serum.

The dialysis outcomes and practice patterns study (DOPPS) showed that pre-dialysis hyperphosphatemia was positively associated with all-cause and cardiocerebrovascular death in MHD patients [17]. However, some studies have provided different opinions. In a retrospective study using a Cox proportional hazards regression model, Fang et al. found that neither baseline nor time-dependent pre-dialysis phosphate concentrations were significantly associated with all-cause and cardiovascular death [18]. A multicenter prospective registry study in Japan [13] found that the mortality rate was unaffected by serum phosphate concentrations after adjustment for confounding factors. Some factors were traditionally-recognized risk factors, such as age, sex, and diabetes. The present study showed that pre-dialysis phosphate concentrations differed significantly between our four groups before matching and that pre-dialysis phosphate concentration was significantly associated with all-cause and cardiocerebrovascular death. After case–control matching, the influence of age, sex, diabetes and pre-dialysis phosphate on mortality rates in the four groups was eliminated. These results suggest that age, sex, diabetes, and other factors have a greater impact on the mortality rate of MHD patients compared with pre-dialysis serum phosphate concentrations, given the confounding effects of the variables.

Although some authors believed that PRR can be similar to URR in evaluating phosphate clearance during dialysis [15], unlike URR, PRR does not closely reflect changes in whole body phosphate mass, as most of the phosphate is retained in the central compartment (intracellular fluid of bone cells) and inaccessible to the intermittent dialysis processes. In addition, phosphates are actively transported from these central compartments to the plasma during and after dialysis, counteracting changes in phosphate concentrations caused by dialysis and leading to significant post-dialysis rebound. Therefore, some studies suggested that adequate conventional hemodialysis (4 h/3 times per week), even in conjunction with dietary phosphorus restriction, may be ineffective in managing hyperphosphatemia [19]. Our research data validates this point: while there are significant differences in PRR among the four patient groups, there is no statistically significant difference in URR values between the groups.

The KDIGO guidelines suggested increasing dialytic phosphate removal in the treatment of persistent hyperphosphatemia in patients with stage 5 CKD [16]. However, some previous studies found that rapid phosphate reduction may have an impact on cell structure and function, and may even increase mortality rates. Lemoine et al. [12] found in an animal study of phosphate kinetics that the decrease in extracellular phosphate stabilized after 3 h of HD, and then intracellular phosphate began to rise approximately 30 min before the end of dialysis. Phosphate is essential for cell signaling activity; However, high concentrations can induce mitochondrial oxidative stress and apoptosis [14]. An imbalance in intracellular and extracellular phosphate concentrations may disrupt the maintenance of signaling activity, even leading to cell and tissue damage [20]. Fang et al. [18] showed that patients with high phosphate clearance during dialysis had high pre-dialysis phosphate concentrations, and the mortality rate was higher in these patients accordingly, especially in patients with normal or low pre-dialysis phosphate concentrations. In other words, the mortality rate of MHD patients was positively correlated with dialytic phosphate removal, but not significantly related to the pre-dialysis serum phosphate concentration. Similarly, our study also found that after matching for sex, age, and diabetes, patients with high PRR during dialysis also had high pre-dialysis phosphate concentrations. However, there were no significant differences in mortality rates between the groups with different dialytic PRR. Our study showed that conventional dialytic PRR were not associated with all-cause mortality or cardiocerebrovascular mortality in MHD patients.

When the KDIGO CKD-mineral and bone disorder guidelines recommended the treatment of hyperphosphatemia by increasing dialytic phosphate clearance, the reference literature showed that frequent nocturnal dialysis could effectively reduce serum pre-dialysis phosphate concentrations. However, there is no evidence that increasing dialytic phosphate removal improves the clinical outcomes of dialysis patients. A previous study also found that frequent nocturnal dialysis reduced serum phosphate concentrations and hypertension during a 12-month observation period; however, no comprehensive benefit was observed owing to vascular access complications and other factors [21]. In principle, higher dialysis frequency or longer dialysis time can remove more phosphate than that with conventional dialysis; however, dialytic phosphate removal accounts for only a small part of the total phosphate in the body and has little effect on the total phosphate content in vivo. Additionally, the abnormal pathophysiological mechanism affecting the exchange of serum phosphate and bone phosphate remains in the uremic state; therefore, the serum phosphate concentration rebounds rapidly after dialysis. However, higher dialysis frequency or longer dialysis time can maintain low or normal serum phosphate concentrations over a relatively long period of dialysis. Additionally, the duration of persistent hyperphosphatemia (dialysis interval) is significantly shorter with these methods compared with conventional dialysis. Further studies are needed to demonstrate whether increasing the frequency of dialysis or prolonging the duration of dialysis can improve the outcomes of MHD patients by maintaining lower serum phosphate concentrations long-term.

This study had the following limitations: First, this was a retrospective cohort study with numerous uncontrollable confounders. Second, some important data, such as dry weight, dialysis mode, dialysis membrane area, and calcium concentration, were not available. Additionally, our findings need to be validated in prospective interventional studies or basic experimental animal models.

## Conclusion

Our study shows that the level of PRR is not related to the mortality rate of patients on routine dialysis three times a week. Whether improving PRR by changing filters or increasing blood flow can improve the prognosis of patients needs further RCT research to verify.

## Data Availability

The datasets generated and analyzed during the current study are not publicly available due to access to data restricted in accordance with government mandates but are available from the corresponding author on reasonable request.

## Abbreviations

MHD: Maintenance hemodialysis
PRR: Phosphate reduction rate
URR: Urea reduction rate
iPTH: Intact parathyroid hormone
CKD: Chronic kidney Disease
HD: Hemodialysis
KDIGO: Kidney Disease: Improving Global Outcomes
DOPPS: Dialysis Outcomes and Practice Patterns Study
SD: Standard Deviation
IQR: Interquartile Range
HR: Hazard Radio
95% CI: 95% Confidence Interval

## References

[1] Liyanage T, Ninomiya T, Jha V, Neal B, Patrice HM, Okpechi I (2015). Worldwide access to treatment for end-stage kidney disease: a systematic review. Lancet.

[2] Wojtaszek E, Oldakowska-Jedynak U, Kwiatkowska M, Glogowski T, Malyszko J (2021). Uremic Toxins, Oxidative Stress, Atherosclerosis in Chronic Kidney Disease, and Kidney Transplantation. Oxid Med Cell Longev.

[3] Chen R, Yang C, Zhu M, Chu H, Wang J, Gao B, et al. Association of cardiovascular disease with 30-day hospital readmission in Chinese patients receiving maintenance dialysis. Ann Transl Med. 2021;9(8):617. 10.21037/atm-20-2367. 10.21037/atm-20-2367 PMC8106029 33987315

[4] Cozzolino M, Mangano M, Stucchi A, Ciceri P, Conte F, Galassi A. Cardiovascular disease in dialysis patients. Nephrol Dial Transplant. 2018;33(suppl_3): iii28-iii34. 10.1093/ndt/gfy174. 10.1093/ndt/gfy174 PMC6168816 30281132

[5] Tiong MK, Ullah S, McDonald SP, Tan SJ, Lioufas NM, Roberts MA, Toussaint ND (2021). Serum phosphate and mortality in incident dialysis patients in Australia and New Zealand. Nephrology (Carlton).

[6] Razzaque MS (2022). Phosphate Metabolism: From Physiology to Toxicity. Adv Exp Med Biol.

[7] Fernández-Martín JL, Martínez-Camblor P, Dionisi MP, Floege J, Ketteler M, London G (2015). Improvement of mineral and bone metabolism markers is associated with better survival in hemodialysis patients: the COSMOS study. Nephrol Dial Transplant.

[8] Song YH, Seo EH, Yoo YS, Jo YI (2019). Phosphate supplementation for hypophosphatemia during continuous renal replacement therapy in adults. Ren Fail.

[9] Pistolesi V, Zeppilli L, Fiaccadori E, Regolisti G, Tritapepe L, Morabito S (2019). Hypophosphatemia in critically ill patients with acute kidney injury on renal replacement therapies. J Nephrol.

[10] Thongprayoon C, Cheungpasitporn W, Hansrivijit P, Thirunavukkarasu S, Chewcharat A, Medaura J (2020). Impact of serum phosphate changes on in-hospital mortality. BMC Nephrol.

[11] zhu M, Dou L, Zhu M, et al. Variability of serum phosphate and its association with death among hemodialysis patients. Clin Nephrol. 2018;90(2):79–86. 10.5414/CN109265. 10.5414/CN109265 29578398

[12] Lemoine S, Fournier T, Kocevar G, Belloi A, Normand G, Ibarrola D (2016). Intracellular Phosphate Dynamics in Muscle Measured by Magnetic Resonance Spectroscopy during Hemodialysis. J Am Soc Nephrol.

[13] Sueta D, Tabata N, Tanaka M, Hanatani S, Arima Y, Sakamoto K (2020). Associations between corrected serum calcium and phosphorus levels and outcome in dialysis patients in the Kumamoto Prefecture. Hemodial Int.

[14] Nguyen TT, Quan X, Hwang KH, Xu S, Das R, Choi SK (2015). Mitochondrial oxidative stress mediates high-phosphate-induced secretory defects and apoptosis in insulin-secreting cells. Am J Physiol Endocrinol Metab.

[15] Hicham R, Taoufiq A. Driss EK. The Impact of Blood Flow Rate on Dialysis Dose and Phosphate Removal in Hemodialysis Patients. Saudi J Kidney Dis Transpl. 2018;29(4):872–8. 10.4103/1319-2442.239654. 10.4103/1319-2442.239654 30152424

[16] Kidney Disease: Improving Global Outcomes (KDIGO) CKD-MBD Update Work Group. KDIGO 2017 Clinical Practice Guideline Update for the Diagnosis, Evaluation, Prevention, and Treatment of Chronic Kidney Disease-Mineral and Bone Disorder (CKD-MBD). Kidney Int Suppl (2011). 2017;7(1):1–59. 10.1016/j.kisu.2017.04.001. 10.1016/j.kisu.2017.04.001 PMC6340919 30675420

[17] Lopes MB, Karaboyas A, Bieber B, Pisoni RL, Walpen S, Fukagawa M (2020). Impact of longer term phosphorus control on cardiovascular mortality in hemodialysis patients using an area under the curve approach: results from the DOPPS. Nephrol Dial Transplant.

[18] Fang YW, Leu JG, Tsai MH, Liou HH. Higher Intra-Dialysis Serum Phosphorus Reduction Ratio as a Predictor of Mortality in Patients on Long-Term Hemodialysis. Med Sci Monit. 2019;25:691–9. 10.12659/MSM.913137. 10.12659/MSM.913137 PMC6354640 30674864

[19] Shaman AM, Kowalski SR (2016). Hyperphosphatemia Management in Patients with Chronic Kidney Disease. Saudi Pharm J.

[20] Razzaque MS (2011). Phosphate toxicity: new insights into an old problem. Clin Sci (Lond).

[21] Rocco MV, Lockridge RS, Beck GJ, Eggers PW, Gassman JJ, Greene T (2011). The effects of frequent nocturnal home hemodialysis: the Frequent Hemodialysis Network Nocturnal Trial. Kidney Int.

